# Supplementary figures and images for: The visual cortex produces gamma band echo in response to broadband visual flicker
Source: PLoS Comput Biol. 2021 Jun 1;17(6):e1009046. doi: 10.1371/journal.pcbi.1009046 (PMC8195374; doi:10.1371/journal.pcbi.1009046)

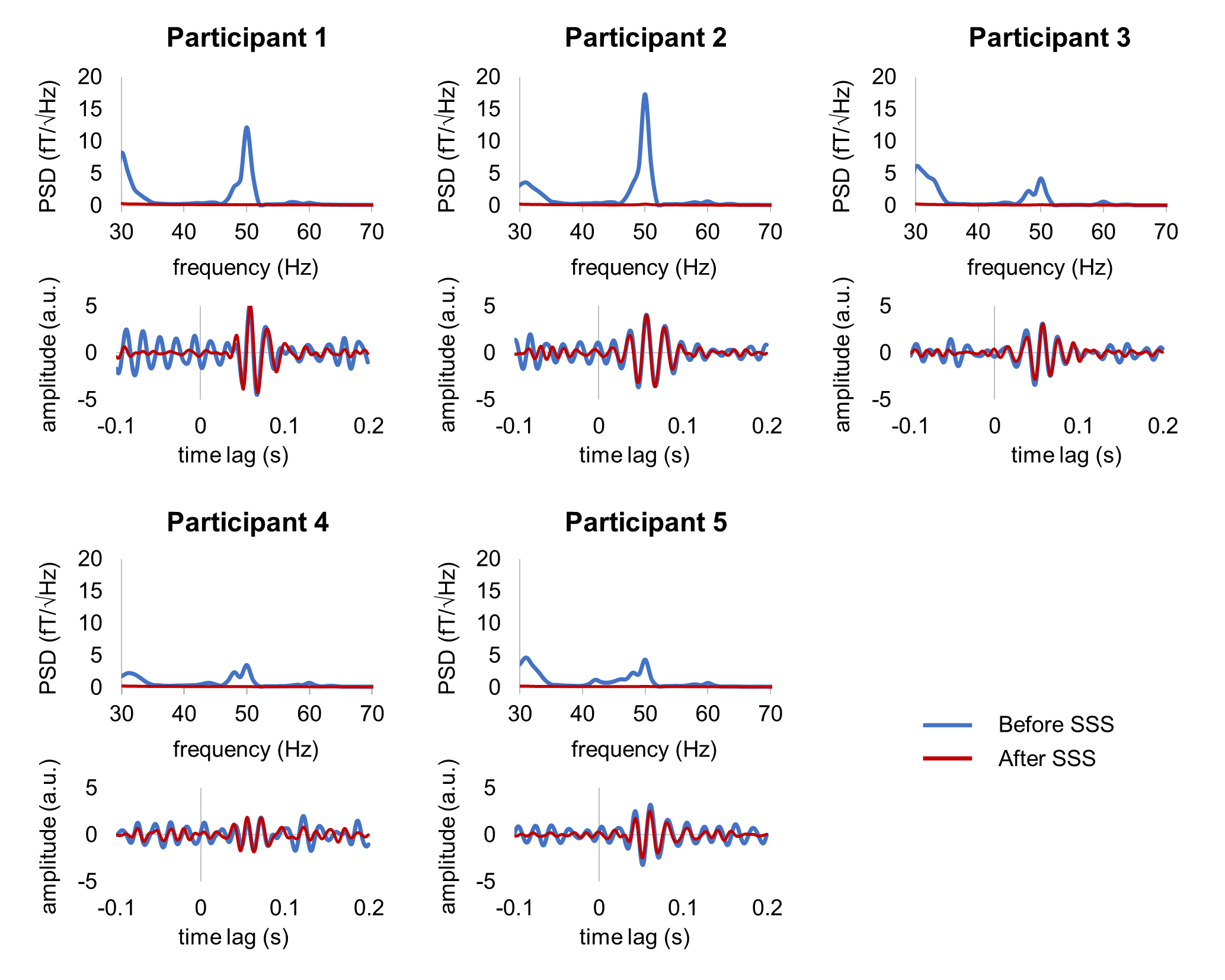

Supplement: S1 Fig — Each panel shows the power spectral density (PSD) and TRF for individual participants before (blue line) and after (red line) applying the SSS method to suppress 50 Hz line noise. The echoes remain strong after the 50 Hz line noise is suppressed. (TIF) [file pcbi.1009046.s001.tif]
