# Supplementary material for: The visual cortex produces gamma band echo in response to broadband visual flicker
Source: PLoS Comput Biol. 2021 Jun 1;17(6):e1009046. doi: 10.1371/journal.pcbi.1009046 (PMC8195374; doi:10.1371/journal.pcbi.1009046)
Supplement: Attachment — Submitted filename: Authors_response_R1.pdf [file pcbi.1009046.s002.pdf]

We thank reviewers for the highly constructive comments. We addressed their concerns to the best of our knowledge and the manuscript is now much improved.

Corrections in the manuscript are highlighted in red.

We have made the data and code available on OSF website (https://osf.io/fe8x5/).

Reviewer #1:

Summary: The authors report a novel oscillatory response function in the gamma band (~48Hz) by stimulating the visual cortex with white noise visual flicker. They also implement a PING model that replicates the same dynamics. Overall the paper is clearly written, and the findings are of interest for neuroscientists investigating oscillatory dynamics. I only have a few minor points that hopefully could help improve the manuscript.

Main points :
- one main concern about the presentation of these results is the lack of representation of the gamma echoes topography. Besides specifying precisely the location of each sensor used in the analysis for each subject, it would be very interesting to show these echoes' cortical extent. Are they localized mostly in occipital regions, or do they also appear in more central/frontal ones? Besides, the PING network does not necessarily model visual areas, and it could be interesting to generalize your results to other sensory (or non-sensory) cortical regions.

- Now we conducted additional analysis to describe topographies of the gamma echo and grating induced gamma oscillations at the sensor and source levels (see, lines 161-174). The results showed that both the gamma echo and induced gamma oscillations originated in the primary visual cortex and did not appear to propagate outside of this area.

- It is not clear to me the difference between endogenous gamma and gamma echoes (and flicker responses). Related to the previous point, would it be possible to localize precisely to V1 the gamma echoes, to support the authors' claim that these are originated in V1, whereas endogenous gamma is originated in V2? Also, what would be the functional role –if any- of gamma echoes and endogenous gamma oscillations? In this light, why would the endogenous gamma be higher in the visual hierarchy (as suggested by the authors)?

- Indeed, the link between induced gamma activity and the gamma echo needed to be better characterized. To address this point, we computed time-frequency representation of MEG power at the occipital sensor with the strongest response to flicker and described the results (see, lines 148-154). In short, the visual grating stimuli induced gamma oscillations at around 57 Hz (grand average), while the frequency of the gamma echo was around 48 Hz. This result together with the source level analysis suggests that the gamma echo and induced gamma oscillations are produced by different but neighboring sources. This is also consistent with our recent work by Duecker et al. (2020; bioRxiv).

- Your simulation shows that an oscillatory input at the right resonance frequency (48Hz) is amplified by the network, producing an amplified peak in the power spectral density. Isn't this result in contradiction with previous findings showing that visual stimulation

may not entrain gamma oscillations? (reference [46] in the paper, from the same senior author as the current study).

- This is an excellent point which prompted us to quantify the induced gamma oscillations (see Fig. 3). In this revised version, we reconsidered and refined parameters of our model, so that the results slightly changed (lines 287-302). In contrast to our previous observations, the amplification was not narrowband but rather peaked at around 48 Hz and decayed towards higher frequencies (see, Fig. 10E). This result closely resembled findings by Gulbinaite et al., 2019 at higher frequencies > 40 Hz (see, Fig. 3C in Gulbinaite et al., 2019). For convenience, we combined Fig. 10E and Fig. 3C from Gulbinaite et al., 2019 into a single figure R1 (see below).

- Indeed, study by Duecker et al., 2020 (old reference [46]) did not report a resonance frequency, which may seem like a contraction to our current findings. However, considering the source modelling results, we conclude that the gamma echo and the induced gamma oscillations are produced by different generators; while the gamma echo is produced by area V1, the endogenous gamma oscillations are produced slightly downstream (possibly area V2). This is now fully consistent with the work of Duecker et al, 2020 demonstrating the visual flicker does not entrain endogenous gamma oscillations. We have updated the Discussion section accordingly (lines 373-390).

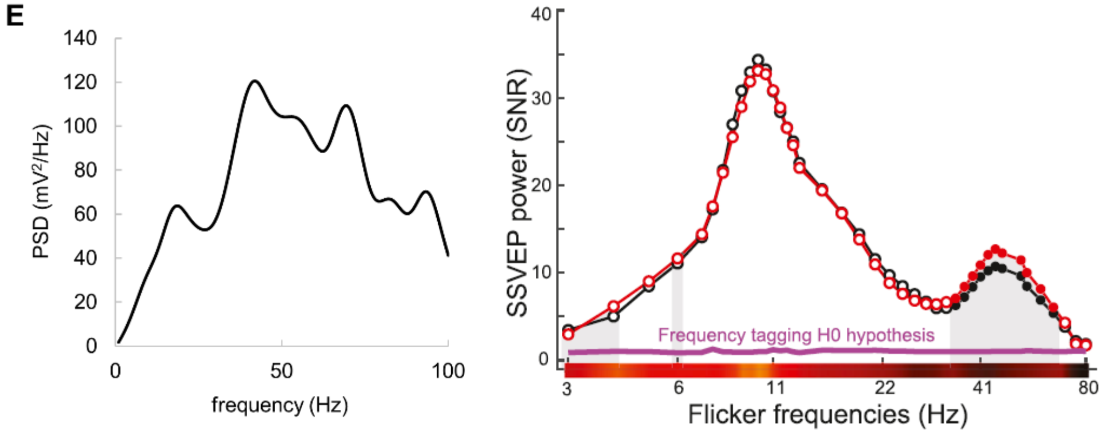

Fig. R1. (*Left panel*) Power spectral density of the model output (mean membrane potential) for oscillatory input currents over multiple frequencies, 1–100 Hz. (*Right panel*) SSVEP at different flicker frequencies (adapted from Gulbinaite et al., 2019; Fig. 3C). Note the logarithmic scale of x-axis on the right panel.

- The model's coefficients and parameters have not been explored at all in this study. However, it could be interesting to investigate how the TFR changes properties as a function of some relevant parameters. Ideally, this could shed some light on the differences between participants (peak frequency, amplitude, etc.). Additionally, it would be best to motivate the choice of the parameters' value (are they all the same as in the cited Izhikevich model ?) and their biological plausibility. Would the coefficients' value be different in other brain regions (e.g., higher in the visual system), thus predicting differences in the TFR?

- We now evaluated different parameters of the model and described the results in corresponding sections. Please see the following lines: 207-221; 230-234; 242-247; 274-281; 312-321; 587-593.

- The exploration of parameters has helped us to ensure that the dynamics of the model is indeed robust. As expected from the literature the GABAergic conductance is a key parameter to set frequency of the network.

- Why equalizing the number of trials per condition in each participant? Isn't it reasonable to assume that the two conditions (coherent and incoherent motion) are orthogonal to the TRF?

- We equalized the number of trials per condition because the resulting TRF is an average of the TRF computed for individual trials. This procedure serves to avoid a potential bias in TRF estimation related to unbalanced number of trials. We added this note in the Method section (lines 497-498).

- The Results section's first paragraph mentions that figure 2B is from an occipital sensor of a representative participant. However, none of the subplots in figure 3 (with all participants) looks like the same image, despite having the same scale.

- Indeed, MEG responses to the flicker depicted in Fig. 2B (now Fig. 1B) and Fig. 3 (top left) (now Fig. 2, top left) were assessed for the same participant (S1), but at slightly different occipital sensors (~4 cm apart). The reason is that the sensor with strongest response to flicker did not capture the alpha echo, so we arbitrarily selected an occipital gradiometer that captured both alpha-band and gamma-band echoes. We updated the text accordingly, “Note that the TRF was computed for an occipital gradiometer that captures both the alpha-band and gamma-band TRF” (line 159-161).

- We have made the data and code available on OSF website (https://osf.io/fe8x5/). The MATLAB script “data/ge_TRF.m” can be used to reproduce the gamma echoes.

- It would be interesting, in the next study, to replicate these results on a larger population. In alpha-band echoes, some participants don't manifest any reverberation, possibly due to the cortex's anatomical configurations. If this is not the case in the gamma echoes (from figure 3, it seems that 5 participants out of 5 show them quite clearly), this could provide some indications that different mechanisms are involved in the two frequency bands.

- There are several differences between alpha-band and gamma-band echoes. First, while alpha echo occurs at around 0.2 s, gamma echo has much earlier onset at 40 ms. Second, gamma echo is largely localized in the primary visual cortex, but alpha echo propagates over the cortex (e.g., Alamia and VanRullen, 2019). Third, the alpha-echo spans for up to 10 cycles, while gamma echo vanishes after 2–3 cycles. Thus, all these different characteristics suggest that the echoes may not be strongly related and perhaps, generated by different mechanisms. We now added this to the Discussion (lines 345-351).

- Related to the previous point, it could be interesting to discuss/speculate about the differences between the echoes in the two frequency bands: do they both reflect two resonance properties of different neuronal networks, the gamma one more locally and

the alpha one more globally? Or do they underlying different mechanisms and functions?

- Now we incorporated these points to the Discussion section under the title "*Relationship between alpha and gamma echoes*" (lines 343-372).

Minor details :
- in the 'power spectral density' the epochs are defined as 5 seconds long; in the previous paragraph, it was 4. Which one is it?

- Indeed, there was a typo in the description and now we corrected it accordingly (lines 501-502). To avoid confusion, in the revised version we made MEG and simulated data trials of equal length (4 s).

- It seems that some figures are referenced in the wrong place. The 'Model' paragraph refers to figure 2A, but the model is in figure 4. Similarly, later the connectivity matrix refers to figure 3A, but I see it in 4B.

- We apologies for this confusion. Now we corrected the figure references as follows: "We modelled the neuronal populations of cortical areas as a network of interconnected excitatory and inhibitory neurons (Fig. 5A)".

Reviewer #2:

In this manuscript the authors applied a broadband flicker (1-720 Hz) to drive human visual cortex while measuring the MEG response. Moreover they estimated the temporal response function (TRF) between the visual input and the MEG response, which revealed an early response in the 40-60 Hz gamma range as well as in the 8-12 Hz alpha band. Simulations of a network model for gamma oscillations with a broadband signal complete the results, thus allowing the authors to estimate numerically the TRF and to compare it with the one from MEG study.

The writing of the article is often sloppy and many paragraphs need to be re-written in order to improve clarity. Some examples are reported in the detailed list of comments/questions in the following.

Moreover I find the Introduction and the Discussion too focused on the experimental results, while a sufficient literature regarding the state-of-art of the models is neglected.

- We now described relevant models in the Introduction (lines 85-93) and Discussion (lines 405-422) sections.

All in all the results of this manuscript are more oriented towards numerical experiments than the experimental ones, thus creating an imbalance in the discussion.

- Now we added more experimental results including source modelling of the gamma echo and induced gamma oscillations, and time-frequency representation of the induced gamma activity at sensor level. These results are crucial for understanding neurophysiological basis of the gamma echo. We hope that the discussion now is more balanced.

In the following is reported a list of comments/questions.

INTRODUCTION

1. Citing the text:
Furthermore, the GABAergic feedback also serves to synchronize the population activity [14,15] "The pyramidal neurons also play an important role in the mechanism." In each cycle [...] The sentence reported in "..." seems to have been forgotten there.

- We removed the redundant sentence.

2. [..] we recorded the MEG while stimulation the visual system using a broadband (1-720 Hz) visual flicker [..] Please replace "while stimulation" with "while stimulating"

- Corrected.

MODEL

3. Why do you use such a limited number of neurons (200 in total)?

- In this revised version, we increased the number of neurons to 500, following methodology in Quax et al., 2017, PLOS Comp Biol. Now we also explored the impact of the network size on the model firing rate (frequency) and power (see, lines 242-247).

4. How is chosen the connectivity matrix?

- In this revised version, we refined the model parameters, so the connectivity has been changed. Detailed description of the connectivity matrix is now provided in the text (lines 587-602).

RESULTS

5. "The temporal response function (TRF) of a system..."
The acronym TRF has been already introduced before; there is no necessity to re-introduce it now.

- Corrected.

6. Merge the paragraphs "TRF show individual resonance frequency of gamma echo" and "The gamma echo is close to 50 Hz but not due to line noise" for a better comprehension of the results shown in Fig. 3. At the moment it is necessary to read the latter paragraph in order to understand what is written in the previous one, where Fig. 3 is cited.

- Now the paragraphs are merged as suggested (see, lines 134-143).

7. Could you better explain the motivations underlying the choise of connection strength? In the text is written: "we used a relatively low connection strength between E-cells (cee = 0.001), high connection strength between I-cells (cii = 0.200), while keeping connectivity between E-to-I-cells (cei = 0.050) and I-to-E-cells (cie = 0.010) at a moderate

level"
A biophysical justification would help.

- We now provided a detailed explanation in the text (lines 587-602).

8. Fig. 4: Merge panels C and D. The information now contained in panel C is negligible. Add in panel A the symbols for 2 external curves impinging both E and I-cells.

- Corrected as suggested (see, Fig. 5).

9. Fig. 4: If you simulate for longer times, do you still see oscillations? Locking at panel E, it seems that the amplitude of the oscillations is shrinking, thus suggesting a possible transient phenomenon.

- We now simulated signal of 4 seconds long, to make it consistent with MEG data. As described in the text (lines 230-234), the transient effect lasted less than 0.5 s, and we discarded the first 1 s of the signal in all the analyses.

10. Fig. 4: A dependence of the results on the amplitude of the external current need to be shown in order to justify the choise of 25 pA.

- Indeed, the amplitude of input currents strongly determine the output characteristics of the model. Now we explored the parameter space of the input currents while preserving the connectivity strength and the network size (lines 207-221).

11. Fig. 5: How much do the results depend on the amplitude of the noise on the E-cells? What happens for smaller and bigger amplitude values with respect to 16pA?

- We now explored the impact of the amplitude of broadband signal on the characteristics of gamma echo (lines 274-281). The results clearly showed that a specific input current (in this case ~4 µA) is required to generate the gamma echo that is comparable with that in MEG. Lower or higher input currents produced responses with incomparably long or short (respectively) decay times.

12. The definition of the external current as "a sinusoidal function with a given frequency (0 - 100 Hz)" is misleading. It should be clearly mentioned the employed value (or values) of the used frequency.

- We improved the description of the oscillatory input (lines 297-299).

13. Fig. 6: In panel C it is calculated the average membrane potential of the E-cells. Is it the same also for the previous figures? If yes, please write it explicitly.

- Yes, all this is now corrected and clarified in the caption.

14. Fig 6: Which is the range of observability of the shown phenomenon? If you vary the frequency of the external current, in which frequency range do you still observe it?

- Now we explored the model response to different oscillatory inputs (lines 312-321) and described the range in which the amplification effect is observed.

Are you able to observe both alpha and gamma peaks at the same time?

- We did not observe both alpha and gamma peaks because the model parameters were tuned to produce gamma band echo. In the Discussion we now write: “In future work it would interesting to extend the model framework such that the network can produce alpha oscillations as well.” (lines 416-417).

From what I see observing panel D, the emergent frequency is simply the frequency of the external stimulation.

- Yes, this observation is correct. Our new results showed that there was a minimum input current of 5 µA, above which, frequency of the network oscillations matched the frequency of the input current. This suggests that the frequency of external stimulation does not directly translate to the firing rate. Now we described this in the text (lines 312-321).

15. Fig. 6: is the process shown in panel C stationary? What does it happen if you simulate for longer times?

- As we discussed earlier (point 10), the transient effect was relatively short (~0.5 s), and we discarded the first 1 s of the signal from the analysis to make sure that the remaining signal is stationary (see, lines 230-234).

DISCUSSION

16. Please replace "Using broadband visual input stimuli were here provide evidence for [..}" with "Using broadband visual input stimuli we here provide evidence for [..]"

- Corrected.

To conclude, the manuscript cannot be published in this form: the text is sloppy and a detailed investigation of the phenomenon is not presented. In order to understand the origin of the phenomenon, a deeper investigation has to be provided. At the moment I just see (probably) transient phenomena, that are shown just for few exemplary cases, without giving a clear view on the dependence on the amplitude and the frequency of the external stimulation.

We now expanded the results section and re-written a large part of the manuscript. Thanks for the constructive comments that now have allowed us to significantly improve the paper.

Reviewer #3:

The authors used a novel visual gratings task where subjects were asked to assess motion congruence/incongruence per trial. The left and right visual stimuli (presented to

the left and right of fixation) were generated such that luminance was varied using orthogonal broadband random signals (i.e. noise with uniform distribution) flickering signals. MEG was recorded in 5 adult subjects. The Temporal response function (TRF) showed a subject specific gamma echo (40-60Hz) as well as an alpha (8-12Hz) echo. The dominant frequency of the novel gamma echo was subject specific and thus likely a reflection of early visual cortex activity. A PING model was designed to then mimic these TRF responses, lending support for contribution of GABA signaling in the early processing of visually presented stimuli.

Impression: The experiment and modeling findings are interesting. I did think that the methods description regarding the visual stimuli could have included more detail.

- We improved description of the methods and the stimuli (lines 456-472).

In addition, the implications of these findings to prior knowledge regarding visual processing could be discussed further (implications wrt perception/ visual acuity/ evoked responses, etc. or new opportunities to evaluate visual impairment, etc. might be discussed).

- Now we added a section in the Discussion with regards to evoked responses in early visual areas (lines 423-431).

As written, we just get the sense that emitted and echoed gamma responses arise from different visual processing areas. How can we use this new information be used to study/explain visual processing?

- Our results on source localization for the gamma echo and induced gamma oscillations (lines 161-174) suggest that the gamma echo and induced gamma oscillations are generated by different sources. The computational model provides a possible explanation on how these two phenomena may coexist in the visual system. Now we added this to the Discussion section under heading “Mechanism of the gamma echo” (lines 391-404).

Q1: Why is it called the “alpha band perceptual echo”? Is conscious perception necessary?

- The term *perceptual echo* is coined by VanRullen and MacDonald (VanRullen and MacDonald, 2012, Curr Biol.). The authors stated that “flicker perception tended to be preceded by an increase of alpha-band (8–15 Hz) stimulus amplitude, compatible with the notion that the perceptual effects of the ~10 Hz echo functions include a subjective impression of flicker”.

Q2. Relatedly, why are you using the term “gamma echo” and not the “gamma perceptual echo”?

- As we mentioned earlier (see, Q1), alpha-echo shown to be accompanied by a subjective impression of flicker (VanRullen and MacDonald, 2012). In this case, the intensity of reported flicker depended on the pre-stimulus [-1.0, -0.5] amplitude of alpha band oscillations. Because gamma band amplitude is much smaller compared to the alpha band and visual flicker at around ~48 Hz is barely visible, it is difficult to establish

"perceptual" relationship between pre-stimulus gamma oscillations and perception of the flicker. Thus, in this study we did not use the term *perceptual*.

Q3. In Figure 1, I can see the luminance values of each gratings stimulus are different [uncorrelated] between left and right grating stimulus; were the stimuli also uncorrelated [random?] on each trial as well?

- Now we added a sentence "The direction of motion (up / down) of the left and right grating stimuli was random in consequent trials (lines 458-459).

Q4. I don't understand how these luminance signals were used to produce the flicker stimuli. Can you give a more detailed description of the methods rather than simply referring to reference [26]?

- We apologize for the unclear description. We now provided a detailed explanation of generation of visual stimuli (lines 456-472) and added the new Fig. 12A that better explains the paradigm. Please also note that the MATLAB scripts implementing this paradigm are now available at the OSF website (https://osf.io/fe8x5/).

Q5. So, what does the brain response look like at the sensor level? Does the brain response usually appear as a 2 dipole field waxing in amplitude in relation to the luminance of the independent stimuli? It reads that there was a single max gradiometer selected per subject. Shouldn't there be one per hemifield? Was the max observed in the TRF based on all trials (i.e., like an FFT?).

- Now we added these results to main text (lines 162-174).

Q6. How did the selected sensor compare over subjects? Same hemisphere? Same Channel?

- We would like to clarify this point. All sensors were from the occipital regions, not necessarily the same hemisphere but with the strongest response to flicker. It is important to note that sensor with the strongest response to flicker varied over subjects and combining responses over subjects at the sensor level may bias the results. Considering this, we did not compare topographies over subjects, but only frequencies of the echoes. However, now we combined responses at source level, where individual anatomies (i.e., MRI scans) were co-registered to a standard MNI template. These results further suggested that the gamma-band echo is generated by the primary visual cortex. We now described the group-level topographies of the gamma echo in sensor and source spaces (lines 162-174).

Q7. Emitted visual gamma oscillations from static gratings are typically localized to pericalcarine cortex (i.e., primary visual cortex; see Gaetz et al., 2011), which is at odds with the statement in the Conclusion: "…that the gamma echo is produced by early visual cortex (most likely V1) whereas the endogenous gamma oscillations are produced slightly down-stream (e.g. V2)".

- Our new results suggest that indeed sources of the induced gamma oscillations and the gamma echo are close to each other spatially but clearly show different dynamics and

spectral properties. We now also cited several studies (lines 383-384) showing that gamma oscillations induced by static and dynamic grating stimuli, are localized in the primary visual cortex. Unfortunately, we did not find information regarding the localization of visual gamma oscillations (rather localization of movement-related gamma) in Gaetz et al., 2011, NeuroImage, and to avoid confusion, we did not cite this paper.
